# Sedation in specialized palliative care: A cross-sectional study

**DOI:** 10.1371/journal.pone.0270483

**Published:** 2022-07-08

**Authors:** Christel Hedman, Aldana Rosso, Ola Häggström, Charlotte Nordén, Carl Johan Fürst, Maria E. C. Schelin

**Affiliations:** 1 Department of Molecular Medicine and Surgery, Karolinska Institutet, Stockholm, Sweden; 2 Division of Palliative Care, Department of Clinical Sciences Lund, Lund University, Lund, Sweden; 3 The Institute for Palliative Care at Lund University and Region Skåne, Lund, Sweden; 4 R&D Department, Stockholms Sjukhem Foundation, Stockholm, Sweden; 5 Division of Geriatric Medicine, Department of Clinical Sciences in Malmö, Lund University, Malmö, Sweden; 6 Unit of Palliative Care Kristianstad, Region Skåne, Kristianstad, Sweden; 7 Unit of Palliative Care Ystad, Region Skåne, Ystad, Sweden; 8 Department of Research and Development, Skåne University Hospital, Lund, Sweden; Qatar University, QATAR

## Abstract

**Background:**

Palliative sedation is used to relieve refractory symptoms and is part of clinical practice in Sweden. Yet we do not know how frequently this practice occurs, how decision-making takes place, or even which medications are preferentially used.

**Objectives:**

To understand the current practice of palliative sedation in Sweden.

**Methods:**

We conducted a retrospective cross-sectional medical record-based study. For 690 consecutive deceased patients from 11 of 12 specialized palliative care units in the southernmost region of Sweden who underwent palliative sedation during 2016, we collected data on whether the patient died during sedation and, for sedated patients, the decision-making process, medication used, and depth of sedation.

**Results:**

Eight percent of patients were sedated. Almost all (94%) were given midazolam, sometimes in combination with propofol. The proportions of sedation were similar in the patient groups with and without cancer. The largest proportion of the sedated patients died in inpatient care, but 23% died at home, with specialized palliative home care. Among the patients with a decision to sedate, 42% died deeply unconscious, while for those without such a decision the corresponding figure was 16%. In only one case was there more than one physician involved in the decision to use palliative sedation.

**Conclusion:**

8% of patients in specialized palliative care received palliative sedation, which is lower than international measures but much increased compared to an earlier Swedish assessment. The level of consciousness achieved often did not correspond to the planned level; this, together with indications of a scattered decision process, shows a need for clear guidelines.

## Introduction

Palliative sedation has been defined by the European Association for Palliative Care (EAPC) as a medical intervention intended to induce decreased or absent awareness to alleviate refractory symptoms, without either prolonging or shortening the natural dying [1,2]. Depending on what patients´ need are, it can be intermittent or continuous as well as light, intermediate, or deep [3]. While the practice has been and continues to be much debated, it is simultaneously being used in practice, and there is ongoing work to update the EAPC definition [4,5].

The frequency of palliative sedation differs markedly around the world and over time, from 3% in Denmark in 2001 to 18% in the Netherlands in 2015. There are also many local studies showing an even wider variation of practice [6,7]. Comparisons between countries and between studies are hampered by not all studies using the EAPC definition of palliative sedation, but a recent review found indications of an increasing trend over time [8]. In Sweden, one of the latest reports is a cross-sectional study from 2009 showing that 1% (n = 22) of palliative patients were given medication for palliative sedation, and half of these achieved sedation [9].

International guidelines emphasize that the decision to start palliative sedation should be made in accordance with the patient’s wishes; or, when this is not possible, in consultation with a surrogate decision-maker [1]. Despite this, consent from patients or relatives is not always obtained [10].

In this study we aimed for a deeper understanding of the current practice of palliative sedation in Sweden, including the frequency, the underlying disease, the symptoms, the medications and the decision-making surrounding this ethically and medically complex issue.

## Materials and methods

All units for specialized palliative care in the southernmost region of Sweden (Region Skåne, population 1.3 million) were invited to take part in this study by reporting all deceased patients, and whether they died under palliative sedation. Eleven units accepted the invitation (four inpatient units and seven home-care units), and one inpatient unit declined. In total, data were collected on 690 patients during the 6 months of the study (May–October 2016), corresponding to 95% of all patients who died at the included units during this time. All the specialized palliative care units including patients have interprofessional teams consisting of at least physicians, nurses, physiotherapists, occupational therapists, and social workers.

For each patient, the treating physician answered a questionnaire regarding the underlying disease, where the patient died (inpatient care/at home), medication in the last 24 hours, overall level of consciousness in the last 24 hours, and whether the patient died under palliative sedation. If the last question was answered in the affirmative, further questions were asked regarding the decision process (who initiated and participated in the discussion, who made the decision), main indication, medication (what drugs and dosage), and result (depth of sedation at different time points, length of sedation period). Depth of sedation was assessed by the responsible physician according to study instructions (mild = easy to wake, intermittent = with periods of being awake and continuous = without periods of being awake) and reported after death. In addition, the planned level of sedation was asked for: mild, intermittent deep or continuous deep. As mentioned above, the objective of this study is exploratory, e.g. to describe current palliative sedation practice in Sweden. Therefore, no statistical hypothesis testing was performed, and data were analysed solely by descriptive statistics using version 14.2 of Stata IC (StataCorp LLC, Texas, USA).

According to Swedish law, data regarding deceased patients are no longer classified as personal data in the legal sense and are thus no longer covered by the ethical review board mandate and are not protected. The data in the study were de-identified. The Swedish ethical review board has given permission to the study (DNR 2016/210) without a formal ethical approval.

## Results

Of the 690 patients, 53 (8%) died during palliative sedation, as assessed by their treating physician (Table 1). Cancer had been diagnosed in 89% of both the sedated patients and the non-sedated patients in the participating units. Sedated patients were younger than non-sedated patients (mean age 71.0 vs. 74.7 years), and male patients were more likely to be sedated (10% of male vs. 7% of female patients). Regarding place of death, 72% of sedated patients and 43% of non-sedated patients died in hospital, and 23% of sedated patients and 42% of non-sedated patients died at home with specialized palliative home care. Among the patients *without* decision to sedate, 15% were nevertheless deeply unconscious during the last 24 hours before death, while 20% were awake; among the patients *with* decision to sedate, 42% were deeply unconscious and none were awake.

**Table 1:** Demographic data on the total patient group as well as patients with and without palliative sedation.

		With palliative sedation (n,%)	Without palliative sedation (n,%)	Total (n, %)
**Number of patients**		53 (8%)	637 (92%)	690 (100%)
**Mean age at death, years (SD)**		71.0 (11.0)	74.7 (11.2)	74.5 (11.3)
**Sex**			
	Female	23 (43%)	325 (51%)	348 (50%)
	Male	30 (57%)	312 (49%)	342 (50%)
**Place of death**			
	Palliative inpatient care	38 (72%)	273 (43%)	311 (45%)
	Advanced home care	12 (23%)	269 (42%)	281(41%)
	Advanced home care in nursing home	3 (6%)	95 (15%)	98 (14%)
**Main diagnosis**			
	Cancer	47 (89%)	565 (89%)	612 (89%)
	Heart-related	2 (4%)	30 (5%)	32 (5%)
	Lung-related	2 (4%)	10 (2%)	12 (2%)
	Neurological	2 (4%)	16 (3%)	18 (3%)
	Other	0 (0%)	16 (3%)	16 (2%)
**Overall consciousness in the last 24 hours**			
	Awake	0 (0%)	129 (20%)	129 (18%)
	Mild	16 (30%)	273 (43%)	289 (42%)
	Intermittently	15 (28%)	134 (21%)	149 (22%)
	Deep	22 (42%)	101 (16%)	123 (18%)
**Dosage of midazolam in the last 24 hours**			
	0–5 mg	6 (11%)	391 (61%)	397 (58%)
	6–10 mg	5 (9%)	82 (13%)	87 (13%)
	11–20 mg	8 (15%)	95 (14%)	103 (15%)
	21–30 mg	7 (13%)	33 (5%)	40 (6%)
	31–40 mg	9 (17%)	11 (2%)	20 (3%)
	41-50mg	3 (6%)	8 (1%)	11 (2%)
	51-60mg	5 (9%)	8 (1%)	13 (2%)
	61-70mg	1 (2%)	1(0%)	2(0%)
	71-144mg	9 (17%)	7 (1%)	16 (2%)
	Total n	53	636	689 (100%)

In 73% of the cases, the planned level of sedation was achieved after 24 hours of sedation and for the last 24 hours of life the planned level if sedation was achieved in 52% of the cases (Table 2).

**Table 2:** Level of sedation/consciousness during the dying phase, as compared to the planned level of sedation, for patients with decision to sedate. The correspondence between the planned level and the achieved level (marked in grey) was 73% for *level of sedation after the first 24 hours from initiation* and 52% for *overall consciousness in the last 24 hours*.

		Planned level of sedation N			
		Mild	Intermittent deep	Continuous deep	Total
**Level of sedation after 24 hours from initiation**					
	Awake	1	1	0	2
	Mild	25	2	0	27
	Intermittent deep	4	3	4	11
	Continuous deep	2	0	9	11
**Overall consciousness during the last 24 hours**					
	Mild	14	1	1	16
	Intermittent deep	8	4	3	15
	Continuous deep	10	2	9	21
**Dosage of midazolam in the last 24 hours**					
	>0–5 mg	1	3	2	6
	6–10 mg	3	1	1	5
	11–20 mg	6	0	1	7
	>20 mg	22	2	10	34

Data were missing for 2 patients regarding *level of sedation after 24 hours from initiation* and for 1 patient regarding *overall consciousness during the last 24 hours*, and *dosage of midazolam in the last 24 hours*.

Focusing only on patients who received palliative sedation, anxiety was the most common indication for sedation (26%), followed by existential distress (21%) and dyspnoea (21%) (Table 3). Midazolam was used as a sedative in 96% of the patients, and propofol was used in 15%, alone or together with midazolam. The median time of sedation was 36 hours (range: 2–504). Decision-making involved the patients in 43% of cases, the relatives in 79% and the team in 81%. In 36% of cases the decision to sedate was made by a physician other than the patient-responsible doctor; usually the physician on-call. Among those where the decision was made by the physician on call the team and/or relative was involved in the decision in all cases. In only one case was there more than one physician involved.

**Table 3:** Characteristics of patients with palliative sedation.

**Duration of sedation (hours)**		**Mean**	**Median**	**SD**		**Min**		**Max**
		67.8	36.0	96.7		2		504
**Main symptom**					**N**		**%**	
	Existential distress				11		20.8	
	Dyspnoea				11		20.8	
	Anxiety				14		26.4	
	Pain				9		17.0	
	Delirium				5		9.4	
	Other				3		5.7	
**Initiative to sedate taken by**								
	Doctor				26		50.0	
	Team				8		15.4	
	Patient themselves				7		13.5	
	Relatives				2		3.9	
	Combinations of the above				9		17.3	
**Discussion before sedation**								
	Relatives, patient, and team				20		37.7	
	Relatives and team				16		30.2	
	Team				7		13.2	
	Relatives				6		11.2	
	Patient, either themself, with team or with relative				3		5.7	
	Another doctor				1		1.9	
**Decision to sedate made by**								
	Patient’s doctor				33		62.3	
	Doctor on call				11		20.8	
	Other doctor				8		15.1	
	Patient’s doctor and another doctor				1		1.9	
**Medication given for sedation (median, range in mg)**								
	Midazolam only (35, 2–144)				44		75.5	
	Propofol only (2160, 45–4270)				2		3.8	
	Midazolam and propofol combined Midazolam (20, 10–35) Propofol (1680, 960–3800)				6		11.3	
	Levomepromazine				0		0	

*Initiative to sedate taken by* and *medication given for sedation had missing data for 1 patient each*.

## Discussion

This population-based study demonstrated that palliative sedation was provided to 8% of the patients, which is an increase compared to previous results from Sweden but lower than figures in international studies [10]. The underlying disease was cancer in almost 90% of the cases, both for sedated and non-sedated patients. Cancer is a common diagnosis within specialized palliative care, but cancer was not over-represented among those sedated. Worldwide, cancer is the most common diagnosis in patients with palliative sedation [2,11]. A shift towards other diagnoses might be needed, as refractory symptoms can be equally common in other diseases [12]. In line with other studies, the main reasons for palliative sedation in our study were anxiety followed by existential distress and dyspnoea [13,14], while pain was a markedly less common indication [8].

Regarding medication, midazolam was used for the majority of patients in our study, which is also internationally the most commonly used drug for palliative sedation due to its fast onset and short duration [15,16]. No patients were sedated with opioids, again in line with other studies [11]. With regard to decision-making, patients and relatives were included in most cases, as recommended by guidelines [1,17,18]. Swedish ethical guidelines further recommend consultation with another physician, preferably a specialist in palliative medicine, and so it was somewhat surprising that a second physician was consulted in only one case in the present study, especially since in a third of the cases the physician making the decision was not the patient-responsible physician. This finding indicates a need for clearer guidelines regarding decision-making [19].

The proportion of patients who died under palliative sedation was 8% (53/690), 4% (12/281) of those in advanced palliative home care, and 12% (38/311) of those in inpatient palliative care. Thus, almost one quarter of all patients receiving palliative sedation died at home, emphasizing that palliative sedation is feasible at home. This is encouraging, since home is the preferred place of death for many patients [20]. Previous literature contains limited and somewhat dated information about palliative sedation at home, but one review found that sedated patients comprised 15–35% of those dying at home [21] and a later prospective study reported a corresponding figure of 14% [22].

We found a discrepancy between planned and achieved level of consciousness, which might partly reflect the “natural” unconsciousness occurring close to death, and partly be due to lack of clinical competence or routines, such as not using a protocol to assess depth of sedation, or changing needs of patients [23].

Our finding that in most cases only one physician decided on sedation emphasizes the need for clear guidelines on decision making. Such guidelines could potentially also increase the participation of patients and relatives in the decision-making. As previous literature indicates regional differences regarding palliative sedation [9], including information on palliative sedation in the national quality registry of palliative care [24] could contribute to more equal treatment in the future.

## Strengths and limitations

Since palliative sedation is only provided at specialized palliative care units, and since 11 of the 12 units in the investigated region chose to participate, the material is close to population-based and thus carries less risk of selection bias. In addition, the material was collected during a short period of time, and any time trend in the practice of palliative sedation is unlikely to have impacted our findings. One limitation is that the data is not nationwide. International research suggests that there may be regional differences in the use of palliative sedation. Another limitation is that the data collection was performed after patients´ death. This could have resulted in misclassification due to the reporting physician not remembering correctly regarding details of medication used or the timing or goals of sedation. In addition, if the physician starting the sedation was a person on call, details of the decision-making process might not have been fully known to the reporting physician.

In conclusion, palliative sedation is possible to implement in the patient’s home. Patient and relative involvement and the correspondence between planned and achieved level of sedation are good but not optimal, meaning further development of clinical guidelines would be appropriate.

## Supporting information

S1 Dataset (XLSX)
